# Correction: Synthesis and model simulation of the hexagonal to circular transition of perovskite cesium lead halide nanosheets by rapidly changing the temperature

**DOI:** 10.1039/d0ra90051f

**Published:** 2020-05-12

**Authors:** Zhong-Hai Lin, Fei Gao, Hong Chen, Jia-Yi Lei, Zhi Yang, Jun-Wei Cai, Ping-Jian Wang, Min-Qiang Wang

**Affiliations:** Key Laboratory of Intelligent Information Processing in Universities of Shandong, Shandong Technology and Business University Yantai 264005 China zhlin@sdtbu.edu.cn; Electronic Materials Research Laboratory (EMRL), Key Laboratory of Education Ministry, International Center for Dielectric Research (ICDR), Xi’an Jiaotong University Xi’an 710049 China

## Abstract

Correction for ‘Synthesis and model simulation of the hexagonal to circular transition of perovskite cesium lead halide nanosheets by rapidly changing the temperature’ by Zhong-Hai Lin *et al.*, *RSC Adv.*, 2020, **10**, 4211–4217, DOI: 10.1039/C9RA10312K.

The authors regret that the name of one of the authors (Min-Qiang Wang) was shown incorrectly in the original article. The corrected author list is as shown above.

The Royal Society of Chemistry apologises for these errors and any consequent inconvenience to authors and readers.

## Supplementary Material

